# Serum selenium and fasting blood glucose: a cross-sectional study in women of different menopause status

**DOI:** 10.1186/s12905-024-03200-1

**Published:** 2024-06-14

**Authors:** Xiao-Man Ma, Ke-Xuan Li, Yu-Miao Guo, Shu-Yi Jiang, Wan-Zhe Liao, Xu-Guang Guo

**Affiliations:** 1 Department of Clinical Laboratory Medicine, Guangdong Provincial Key Laboratory of Major Obstetric Diseases, Guangdong Provincial Clinical Research Center for Obstetrics and Gynecology, The Third Affiliated Hospital of Guangzhou Medical University, Guangzhou, 510150 China; 2 Department of Clinical Medicine, The Third Clinical School of Guangzhou Medical University, Guangzhou, 511436 China; 3 Guangzhou Key Laboratory for Clinical Rapid Diagnosis and Early Warning of Infectious Diseases, King Med School of Laboratory Medicine, Guangzhou Medical University, Guangzhou, 510000 China

**Keywords:** Serum selenium, Fasting blood glucose, Menopause, T2D, Cross-sectional study

## Abstract

**Background:**

This cross-sectional study aims to explore whether there exists an interaction between selenium and menopause concerning type 2 diabetes (T2D) prevalence and its related indicators such as fasting blood glucose (FBG) and homeostasis model assessment of insulin resistance (HOMA-IR).

**Methods:**

150 women aged 35–60 years old were finally analyzed in this study. Multivariate linear or logistic regression modeling was conducted to explore the association of selenium and the prevalence of T2D besides its related indicators. Subgroup analyses were conducted based on menopause status to assess the potential impact on the relationship.

**Results:**

In the fully adjusted model, serum selenium was positively associated with FBG (β: 0.03, CI: 0.01–0.05) and the prevalence of T2D (OR: 1.04, CI: 1.00–1.08). After stratifying the data by menopause status, compared with the postmenopausal women group, as the serum selenium concentrations increased, the FBG concentrations were significantly higher in the premenopausal women group (*p* for interaction = 0.0020).

**Conclusions:**

The present study found serum selenium was positively associated with FBG and the prevalence of T2D. Furthermore, the relationship between serum selenium and FBG was different in the premenopausal and postmenopausal women. More studies are still needed in the future to verify the relationship as well as to explore the specific mechanisms.

## Background

Type 2 diabetes is a serious and common chronic disease [1], and its complications constitute a major global public health issue [2]. Hence, preventing type 2 diabetes (T2D) and its complications is crucial for protecting people’s health. In this regard, identifying the nutritional components associated with the progression of diabetes plays an important role.

Selenium is an essential element for mammals and plays an important role in human health [3]. It is an antioxidant that can help combat the damage of free radicals and protect cells from oxidative stress [4]. In addition, selenium is also involved in regulating immune system function, DNA and protein synthesis, and other biochemical processes [5]. The evidence linking selenium to glucose metabolism is conflicting. Observational studies have suggested that individuals with T2D exhibit lower levels of selenium and/or glutathione peroxidase 3 (GPx3) activities compared to other healthy subjects [6, 7]. Given that diabetic patients often experience oxidative stress and chronic inflammation [2], it appears that higher serum selenium levels may play a crucial role in mitigating oxidative stress and inflammatory responses, thereby potentially protecting against insulin resistance and T2D, as observed in the EVA study [8]. However, multiple clinical and animal experiments have shown that both inadequate and excessive selenium intake may increase the risk of T2D [9–13]. After a 7.7-year follow-up, supplementation with selenium (200 μg per day) was associated with an increased risk of T2D in 1312 participants from the southeastern USA [14]. High selenium intake may elevate the risk of T2D due to its impact on insulin signaling [15]. A correlation was observed between increased erythrocyte glutathione peroxidase 1 (GPx1) activity and insulin resistance in pregnant women [16]. These conflicting conclusions may hinge on the baseline status [5]. Selenium levels vary significantly across different regions of the world [5]. Data from European countries showed a mean serum selenium level of 85.6 μg/L, whereas in US residents, it was 137 μg/L [13, 17].

The menopausal transition is an impactful period in women’s lives accompanied by metabolic changes. One prominent change after menopause is reducing secretion of the ovarian hormones estrogen and progesterone [18]. Multiple studies have reported an interaction between estrogen and selenium, which may impact the metabolism and utilization of selenium through changes in estrogen levels, as well as the influence of selenium supply and function on estrogen synthesis and metabolism [19]. There was a high correlation of total selenium and Selenoprotein P (SELENOP) concentrations in elderly women, but not in young women [20]. The impact of menopause, as well as exogenous estrogen and progestogens, should be taken into account when examining the relationship between selenium and T2D. However, there is a noticeable scarcity of clinical research investigating the association between selenium and T2D prevalence while considering changes in menopausal status among women. Given the interplay between selenium, T2D, and female hormones, it is imperative to explore potential interactions among these three factors. Therefore, this cross-sectional study aims to explore whether there exists an interaction between selenium and menopause in relation to T2D prevalence and its related indicators such as fasting blood glucose (FBG) and homeostasis model assessment of insulin resistance (HOMA-IR).

## Methods

### Data sources and study population

This cross-sectional analysis included female participants between the ages of 35 and 60 (*n* = 3898) who took part in the three cycles of the National Health and Nutrition Examination Survey (NHANES) from 2011–2012, 2013–2014, and 2015–2016. NHANES is conducted by the Center for Disease Control and the National Center for Health Statistics and employs a complex, multistage, probability-sampling procedure to generate nationally representative estimates of the health and nutritional status of non-institutionalized US residents. The NHANES protocol was approved by the National Center for Health Statistics (NCHS) Research Ethics Review Board, and informed consent was obtained from all participants included in the study. All data supporting the findings of this study are available by the public through the Centers for Disease Control and Prevention website at https://wwwn.cdc.gov/nchs/nhanes/Default.aspx.

**Fig. 1:**
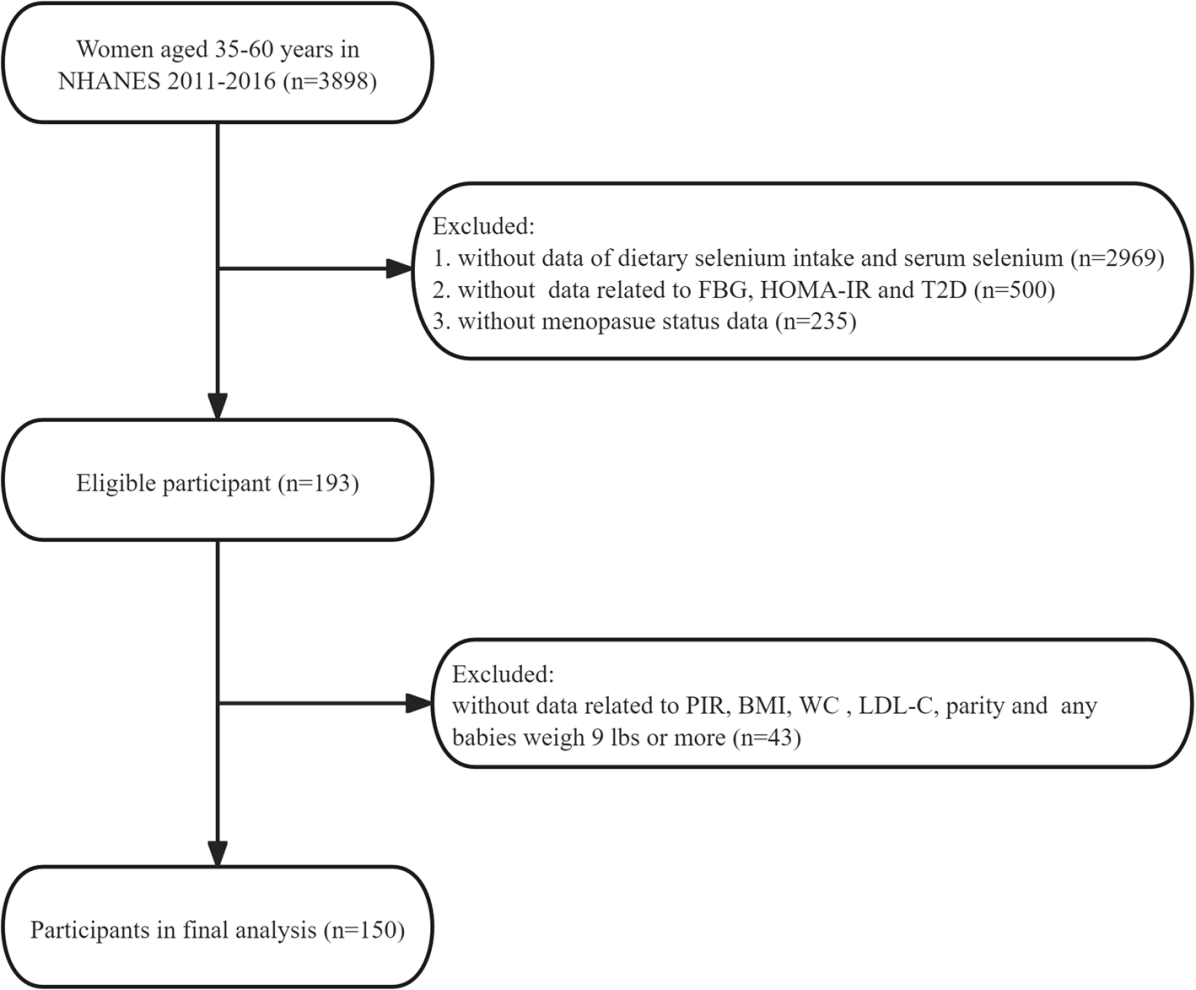
Flowchart of participants enrollment

Individuals were excluded from our analysis if they had missing data for (i) dietary selenium intake and serum selenium; (ii) FBG, HOMA-IR, and T2D; (iii) menopausal status. Furthermore, after excluding individuals with missing data for covariates such as the ratio of family income to poverty (PIR), body mass index (BMI), waist circumference (WC), LDL-cholesterol (LDL-C), parity and any babies weigh 9 lbs or more, 150 participants were finally available for analysis (Fig. 1).

### Selenium assessment and measurement

The assessment of dietary selenium intake in NHANES was conducted using two reliable 24-h dietary recall interviews. Dietary recall interviews are conducted in person by trained dietary interviewers fluent in Spanish and English. And a standard set of measuring guides are used to help the respondent report the volume and dimensions of the food items consumed. The first interview took place at the mobile examination center (MEC), while the second interview was conducted via telephone between 3 and 10 days later. In this study, the final assessment of dietary selenium intake (ug) was obtained by averaging the two measurements of selenium intake. The 24-h recall method is widely used for assessing dietary intake in large-scale surveys. The consistent use of this method in NHANES over the years is supported by expert consensus. Regular workshops are held to evaluate data collection methods in NHANES, and the decision to continue using the 24-h recall method has been made through collaborative discussions.

Serum selenium was measured in whole blood using inductively coupled plasma dynamic reaction cell mass spectrometry (ICP-DRC-MS) that monitored the ion intensity at m/z 80 (80Se). Detailed information about the laboratory method used for selenium measurement can be found in the NHANES documentation provided at this link: https://wwwn.cdc.gov/nchs/data/nhanes/2011-2012/labmethods/cusezn_g_met_serum_elements.pdf.

### Definition of FBG, HOMA-IR, T2DM

FBG (mmol/L) was measured enzymatically in the serum using a Roche/Hitachi Cobas C Chemistry Analyzer, through a hexokinase-mediated reaction. HOMA-IR was calculated using the following formula: fasting insulin (μU/L) × fasting glucose (mmol/L)/22.5. Participants were classified as T2D cases if they met any of the following criteria: (1) Have you ever been informed by a doctor or health professional that you have diabetes or sugar diabetes? (2) HbA1c > 6.5%; (3) FBG ≥ 7.0 mmol/L; (4) Random blood glucose ≥ 11.1 mmol/L; (5) Two-hour OGTT blood glucose ≥ 11.1 mmol/L; (6) Use of diabetes medication or insulin; (7) Type 1 diabetes was excluded.

### Definition of menopause status

Menopausal status was determined by assessing responses to the reproductive health questionnaire. Specifically, women were asked the question, “Have you had at least one menstrual period in the last 12 months, excluding bleeding caused by medical conditions, hormone therapy, or surgeries?” If subjects answered “no” to this question, they were further asked, “What is the reason for not having a period in the last 12 months? (Options: pregnancy, breastfeeding, hysterectomy, menopause/change of life, other)”. Consequently, subjects were classified as postmenopausal women if they reported no menstrual period in the past 12 months and attributed it to natural menopause. Subjects who did not meet the above definition of postmenopausal women were classified as premenopausal women.

### Covariates

Demographic data encompassed age, race/ethnicity (categorized as non-Hispanic White, non-Hispanic Black, Mexican American, and other races), educational level (classified as less than high school, high school, and more than high school), and the ratio of family income to poverty (PIR). To account for the seasonal effect on daily product intake, we added six-month period as a covariate, categorized as 1 November to 30 April and 1 May to 31 October, based on when the examination was performed. In light of the correlation between obesity and T2D or selenium metabolism, covariates such as body mass index (BMI, kg/m^2^), waist circumference (WC, cm), LDL-C (mg/dL), total cholesterol (TC, mg/dL), and dietary intake variables including energy (kcal) and total fat (gm) were incorporated. Furthermore, considering the potential correlation between obstetric and gynecological history and T2D or menopausal status in women, covariates such as parity (categorized as 1–2, 3–5, ≥ 6), gestational diabetes, oral contraceptive use, female hormone use, and having babies weighing 9 lbs or more were also included in this study.

### Statistical analysis

All continuous variables of the present study were approximate symmetric and followed normal distribution. These variables were presented as mean and standard deviation (SD), while categorical variables were expressed as weighted percentages (%). Based on menopause status, participants were categorized as premenopause group and postmenopause group. To determine if there is a significant difference between two groups, the chi-square test was used to analyze when the variables were categorical variables and the t-test was used to analyze when the variables were continuous data.

To explore the association between selenium and the outcomes variables, the multivariate linear regression modeling was employed to estimate β value and 95% confidence intervals (CI) when the outcomes variables were continuous data such as FBG and HOMA-IR, while the multivariate logistic regression modeling was employed to estimate odds ratios (OR) and 95% CI when the outcomes variables were categorized data such as T2D. Three sequential models were used to control for potential confounders. Model 1 did not include any adjustments. Model 2 was adjusted for age and race/ethnicity. Model 3 was further adjusted for age, race/ethnicity, education level, PIR, six-month period when the examination was performed, BMI, WC, LDL-C, TC, energy, total fat, parity, gestational diabetes, oral contraceptive use, female hormone use, and having babies weighing 9 lbs or more. In addition, subgroup analyses were performed based on menopause status and the effect interactions were evaluated using the likelihood ratio test.

Statistical analyses were performed using the R® software package (v.4.2.0, http://www.r-project.org, accessed on 22 April 2022) and Empower® software (v.4.2, http://www.empowerstats.com, X&Y Solutions, Inc. Boston, MA, USA). 95% CI was calculated, and a *p*-value of less than 0.05 was considered statistically significant.

## Results

### Baseline characteristic of participants from 2011–2016

After excluding missing data from women aged 35–60 years in NHANES 2011–2016, a total of 150 women were included in the final analysis. The flowchart detailing the exclusion criteria is summarized in Fig. 1. The baseline characteristics of the participants were displayed in Table 1 according to menopause status. Compared to postmenopausal women, premenopausal women were younger and had a lower education level. Additionally, premenopausal women had higher values of BMI, WC and HOMA-IR. There were no significant differences detected in race/ethnicity, PIR, LDL-C, TC, serum selenium, dietary intake, any of history of obstetrics and gynecology, FBG, and the prevalence of T2D.

**Table 1:** Baseline characteristic of participants from 2011–2016

Covariates	Total	Menopause status		*P*-value
		Premenopause	Postmenopause	
N	150	48	102	
Age (years), Mean ± SD	52.7 ± 6.2	49.7 ± 7.5	54.1 ± 4.9	< 0.001
Race/Ethnicity, n (%)				0.530
Non-Hispanic White	60 (40.0%)	21 (43.8%)	39 (38.2%)	
Non-Hispanic Black	29 (19.3%)	11 (22.9%)	18 (17.6%)	
Mexican American	16 (10.7%)	3 (6.2%)	13 (12.7%)	
Other Race	45 (30.0%)	13 (27.1%)	32 (31.4%)	
Education Level, n (%)				0.028
Less than high school	31 (20.7%)	15 (31.2%)	16 (15.7%)	
High school	26 (17.3%)	4 (8.3%)	22 (21.6%)	
More than high school	93 (62.0%)	29 (60.4%)	64 (62.7%)	
PIR, Mean ± SD	2.6 ± 1.7	2.5 ± 1.8	2.6 ± 1.6	0.666
Six-month period when the examination was performed, n (%)				0.575
1 November through 30 April	70 (46.7%)	24 (50.0%)	46 (45.1%)	
1 May through 31 October	80 (53.3%)	24 (50.0%)	56 (54.9%)	
BMI (kg/m2), Mean ± SD	29.8 ± 7.4	32.2 ± 8.9	28.7 ± 6.2	0.020
WC (cm), Mean ± SD	98.7 ± 16.0	103.7 ± 18.2	96.4 ± 14.4	0.020
LDL-C (mg/dL), Mean ± SD	123.4 ± 37.6	125.0 ± 30.0	122.6 ± 40.8	0.570
TC (mg/dL), Mean ± SD	205.5 ± 41.9	204.0 ± 35.2	206.3 ± 44.9	0.832
Serum selenium, ug/L	129.2 ± 18.3	128.3 ± 16.9	129.6 ± 18.9	0.287
Dietary intake, Mean ± SD				
Energy (kcal)	1740.2 ± 644.3	1833.4 ± 776.3	1696.3 ± 570.9	0.339
Total fat (gm)	68.1 ± 31.8	73.1 ± 36.7	65.8 ± 29.1	0.235
Selenium (ug)	94.8 ± 40.6	98.5 ± 48.1	93.0 ± 36.7	0.756
History of obstetrics and gynecology				
Parity, n (%)				0.097
1–2	54 (36.0%)	20 (41.7%)	34 (33.3%)	
3–5	85 (56.7%)	22 (45.8%)	63 (61.8%)	
≥ 6	11 (7.3%)	6 (12.5%)	5 (4.9%)	
Gestational diabetes, n (%)				0.389
No	133 (88.7%)	41 (85.4%)	92 (90.2%)	
Yes	17 (11.3%)	7 (14.6%)	10 (9.8%)	
Oral contraceptive use, n (%)				0.844
No	36 (24.0%)	12 (25.0%)	24 (23.5%)	
Yes	114 (76.0%)	36 (75.0%)	78 (76.5%)	
Females hormone use, n (%)				0.055
No	109 (72.7%)	30 (62.5%)	79 (77.5%)	
Yes	41 (27.3%)	18 (37.5%)	23 (22.5%)	
Any babies weigh 9 lbs or more, n (%)				0.570
No	121 (80.7%)	40 (83.3%)	81 (79.4%)	
Yes	29 (19.3%)	8 (16.7%)	21 (20.6%)	
FBG, mmol/L	6.2 ± 2.1	6.5 ± 2.8	6.0 ± 1.7	0.554
HOMA-IR	3.5 ± 3.1	4.0 ± 3.2	3.3 ± 3.0	0.049
T2D, n (%)				0.844
No	114 (76.0%)	36 (75.0%)	78 (76.5%)	
Yes	36 (24.0%)	12 (25.0%)	24 (23.5%)	

*PIR* Ratio of family income to poverty, *BMI* Body Mass Index, *WC* Waist Circumference, *LDL-C* LDL-cholesterol, *TC* Total Cholesterol, *FBG* Fasting Blood Glucose, *HOMA-IR* Homeostasis Model Assessment of Insulin Resistance, *T2D* Type 2 Diabetes

### Association between selenium and FBG, HOMA-IR, and T2D

A multivariate regression model was conducted to investigate the association between selenium and FBG, HOMA-IR, and T2D. The results, shown in Table 2, indicated that dietary selenium intake was not significantly associated with any of the outcome variables in all models (*p* > 0.05). However, serum selenium was positively associated with FBG (β: 0.03, CI: 0.01–0.05) and the prevalence of T2D (OR: 1.03, CI: 1.01–1.05) in model 1. These positive associations remained significant after further adjustments for covariates in either model 2 ([β: 0.03, CI: 0.01–0.05], [OR: 1.03, 1.01–1.06], respectively) or model 3 ([β: 0.03, CI: 0.01–0.05], [OR: 1.04, 1.00–1.08], respectively).

**Table 2:** Association between selenium and FBG, HOMA-IR, and T2D

	Model 1		Model 2		Model 3	
	β/OR (95%CI)	*P*-value	β/OR (95%CI)	*P*-value	β/OR (95%CI)	*P*-value
Dietary selenium						
FBG	-0.00 (-0.01, 0.01)	0.4748	-0.00 (-0.01, 0.01)	0.6876	-0.00 (-0.02, 0.01)	0.4718
HOMA-IR	0.01 (-0.00, 0.02)	0.1677	0.01 (-0.00, 0.02)	0.1580	0.00 (-0.01, 0.02)	0.7852
T2D	1.00 (0.99, 1.00)	0.3216	1.00 (0.99, 1.01)	0.5483	1.00 (0.98, 1.02)	0.9305
Serum selenium						
FBG	0.03 (0.01, 0.05)	0.0009	0.03 (0.01, 0.05)	0.0044	0.03 (0.01, 0.05)	0.0010
HOMA-IR	0.01 (-0.02, 0.03)	0.6481	-0.01 (-0.02, 0.03)	0.7193	0.01 (-0.02, 0.03)	0.5057
T2D	1.03 (1.01, 1.05)	0.0075	1.03 (1.01, 1.06)	0.0119	1.04 (1.00, 1.08)	0.0273

Model 1 adjusted for: none

Model 2 adjusted for: age, race/ethnicity

Model 3 adjusted for: age, race/ethnicity, education level, PIR, six-month period when the examination was performed, BMI, WC, LDL-C, TC, energy, total fat, parity, gestational diabetes, oral contraceptive use, females hormone use, any babies weigh 9 lbs or more

*PIR* Ratio of family income to poverty, *BMI* Body Mass Index, *WC* Waist Circumference, *LDL-C* LDL-cholesterol, *TC* Total Cholesterol, *FBG* Fasting Blood Glucose, *T2D* Type 2 Diabetes

### Menopause status modifies the association between serum selenium and FBG

The results in Table 3 showed that in the premenopausal women group, as the serum selenium concentrations increased, the FBG concentrations were significantly higher (*p* < 0.0001). But there was no significant association between serum selenium and FBG in postmenopausal women. Importantly, the interaction of menopause status on the association was significant (*p* for interaction = 0.0020). No significant interaction was observed in the association between serum selenium and HOMA-IR or the prevalence of T2D. No significant interaction was also observed in the association between selenium intake and the three outcomes.

**Table 3:** Interactive effect of menopause and selenium on FBG, HOMA-IR, and T2D

	Premenopause (*n* = 48)		Postmenopause (*n* = 102)		*p* for interaction
	OR(95%CI)	*P* value	OR(95%CI)	*P* value	
Dietary selenium					
FBG	-0.01 (-0.02, 0.01)	0.2879	-0.00 (-0.02, 0.01)	0.7656	0.3849
HOMA-IR	-0.00 (-0.02, 0.02)	0.9431	0.00 (-0.01, 0.02)	0.6682	0.6461
T2D	1.00 (0.98, 1.03)	0.9440	1.00 (0.98, 1.02)	0.8024	0.7797
Serum selenium					
FBG	0.07 (0.04, 0.10)	< 0.0001	0.02 (-0.00, 0.03)	0.1315	0.0020
HOMA-IR	0.02 (-0.03, 0.06)	0.4490	0.01 (-0.02, 0.03)	0.7136	0.6133
T2D	1.06 (1.00, 1.13)	0.0537	1.03 (0.99, 1.07)	0.1235	0.4051

Adjusted for: age, race/ethnicity, education level, PIR, six-month period when the examination was performed, BMI, WC, LDL-C, TC, energy, total fat, parity, gestational diabetes, oral contraceptive use, females hormone use, any babies weigh 9 lbs or more

*PIR* Ratio of family income to poverty, *BMI* Body Mass Index, *WC* Waist Circumference, *LDL-C* LDL-cholesterol, *TC* Total Cholesterol, *FBG* Fasting Blood Glucose, *T2D* Type 2 Diabetes

## Discussion

In this sample of U.S. women aged 35–60 years from 2011–2016, our findings indicate that serum selenium was positively associated with FBG concentration and prevalence of T2D, but non-significantly associated with HOMR-IR. In addition, dietary selenium was not significantly associated with FBG, HOMR-IR, or prevalence of T2D. After dividing the study population according to menopausal status, we found that serum selenium was significantly positively associated with FBG in premenopausal women but not significantly in postmenopausal women. However, there was no significant difference in serum selenium concentrations between pre- and post-menopausal participants. It may suggests that the influence of menopausal status on the relationship is not directly mediated by serum selenium concentrations.

In our findings, selenium was a risk factor for elevated fasting glucose concentrations and prevalence of T2D. However, in some studies, selenium is a protective factor in the development and progression of T2D [1, 21]. This is due to a different baseline for selenium, which has been shown to have a U-curve relationship with the risk of T2D [3], i.e., selenium supplementation in a selenium-deficient state reduces the risk of diabetes, whereas additional supplementation in a selenium-sufficient state increases the risk of diabetes. The U.S. population is selenium-sufficient, and thus selenium showed a negative effect in our study [22].

A multivariate logistic regression analysis of 3406 U.S. subjects showed a positive correlation between a 10 μg/L increase in selenium and a 12% increase in the prevalence of diabetes mellitus [23]. What’s more, high serum selenium levels were also found to be positively associated with the risk of diabetes in a case–control study nested in a prospective cohort [24] and in several cross-sectional analyses [12, 25]. In addition, meta-analyses of experimental and non-experimental studies showed that selenium intake increased the risk of diabetes [26–28]. These conclusions support our results. However, a cross-sectional study of 4106 highly educated young adults in Brazil showed no significant correlation between dietary selenium intake and the prevalence of T2D [29]. In the present study, there was no significant association found between dietary selenium intake and T2D in women. Similarly, in our previous study, this association was also noted, but upon further analysis, we surprisingly discovered an inverted U-shaped relationship in women, with an inflection point of 109.90 μg [30]. When selenium intake was below 109.90 μg, a consistently significant positive association with T2D was observed. However, when selenium intake exceeded 109.90 μg, an inverse association with T2D was noted, although it did not reach statistical significance [30]. A study by Barbara R Cardoso et al. [31] showed a lack of evidence for an association between selenium and diabetes prevalence, but after adjusting for confounders, found that an increase in selenium of 10 μg/L was associated with an increase in insulin of 1.5% (95% CI: 0.4–2.6%) and an increase in HOMA-IR of 1.7% (95% CI: 0.5–2.9%) were associated, i.e., confirming the idea that additional selenium supplementation in populations with high dietary selenium intake is discouraged.

Selenium has been widely studied as an antioxidant, and according to Karalis DT [21], selenium may exert its anti-diabetic effects through antioxidant action. However, it has a narrow therapeutic range and may even cause an increase in FBG concentration and diabetes risk through several mechanisms. It has been found that high dietary selenium intake may result in impaired insulin sensitivity as well as overexpression of GPx1 leading to dysregulated insulin secretion and hyperinsulinemia [32]. In addition, excessive selenium exposure can lead to hepatic insulin resistance by inducing reverse ROS regulation as well as causing excessive activity of antioxidant enzymes, including selenoenzymes, which can affect insulin secretion by interfering with key redox signaling [33].

In the present study, we found that menopausal status had an interaction effect on serum selenium with FBG concentration. Additionally, premenopausal women had higher values of BMI, WC compared with postmenopausal women in the study. A systematic review and meta-analysis concluded that the main change of selenium status is a decrease in GPx activity in the conditions of excess adiposity, particularly in adults with obesity [34]. Considering the association between selenium metabolism and adiposity, BMI and WC were both adjusted as covariates. The results remained stably significant. One prominent change before and after menopause is the difference in estrogen levels. Estrogen status can affect the process of selenium metabolism [19, 35, 36]. Estrogen administration was found to significantly increase erythrocyte GPx, non-GPx, and total activity in both premenopausal and postmenopausal women [35]. Furthermore, plasma selenium levels and GPx1 activity in premenopausal women varied with fluctuations in estrogen levels [19]. This implies that estrogen in the presence of excess selenium exposure may contribute to the negative effect of selenium on T2D risk. There are also possible mechanisms by which, on the one hand, estrogen-mediated increase in GPx1 mRNA in the liver may increase GPx1 synthesis and thus GPx1 activity in females rats [36]; on the other hand, in the same study [36], estrogen treatment may bind to the estrogen response element (ERE) in the promoter region in the selenoprotein P (Sepp1) gene to increase the synthesis of Sepp1.

Our study has several strengths, firstly we chose a very precise and small study population. Secondly, our study possesses the innovation of finding the effect of menopausal status on the relationship between selenium and FBG. Unfortunately, the exact mechanism remains unclear. However, this study also has some limitations. Firstly, it is a cross-sectional study that could not explain the causal relationship of menopausal status with selenium and FBG, and follow-up longitudinal studies are needed to establish a causal relationship. Secondly, the choice of the 24-h recall method for intake assessment is still a topic of significant debate, as it may be subject to dietary recall bias. However, any dietary analysis method commonly used in clinical research, including both 24-h recalls and food frequency questionnaires (FFQs), can produce results that may not accurately reflect the participants’ actual diets. Thirdly, it’s important to note that the study was conducted with a small sample size and within the US population. It should be emphasized that selenium levels vary considerably across different regions globally and the association observed was linked to baseline selenium levels. Finally, due the limitation of small sample size, the present study failed to categorize subjects based on normal/reduced/exceeded selenium blood concentrations to compare the effect in these groups. Therefore, it’s essential not to overestimate our results, as they may not be applicable to populations with differing baseline selenium levels and there was no comparison of patients with normal/reduced/exceeded selenium levels. Larger sample sizes and larger studies involving different ethnic groups are needed to reveal consistent associations.

## Conclusions

Our findings suggest that high selenium exposure is positively associated with FBG and the prevalence of T2D, whereas the relationship between serum selenium and FBG was different in the premenopausal and postmenopausal women. More studies are still needed in the future to verify the interaction of menopausal status on the relationship between selenium and FBG as well as to explore the specific mechanisms.

## Data Availability

All data supporting the findings of this study are available by the public through the Centers for Disease Control and Prevention website at https://wwwn.cdc.gov/nchs/nhanes/Default.aspx.
